# The growth and increase of the number of journals focusing on *Spine*: a scientific blessing?

**DOI:** 10.1007/s00586-014-3254-6

**Published:** 2014-02-26

**Authors:** Max Aebi, Marek Szpalski

**Affiliations:** 1 Orthopaedic Department, Hislanden Salem Hospital, 25, 3000 Bern, Switzerland; 2 Department of Orthopedics, Iris South Hospitals, 1180 Brussels, Belgium

The last 25 years have demonstrated an unmatched growth of knowledge in spinal sciences. Accordingly, this increase of knowledge is reflected not only in an increasing number of journals focusing exclusively on *Spine*, but also in an increase of the number of articles and the number of pages published per journal by a factor of more than ten as well as a continuation of the publication of spine-related articles in the established orthopaedic and neurosurgical journals. In 1976, the first journal focusing only on spine was founded (*Spine*). Later, in 1988, this journal had an offspring in form of the *Journal of Spinal Disorders and Techniques*, before the *European Spine Journal* was founded in 1991. Since then there are more than 15 journals focusing on spine on the market and more may come.

The most probable reason for the massive expansion of scientific communication in the spinal world is the rise of the new technology, originally initiated by surgeons and physicians who were looking for solutions for their patient’s clinical problems and later on rather driven by the Medtech industry offering “solutions” to spine specialists.

But the amount of knowledge is also driven by establishing specialized basic and clinical research, resulting in the researcher’s need of publishing his/her results. Have scientific results been published more frequently in established journals of orthopedic and neurosurgery until the end of the twentieth century, more specialized spine journals started to become active in the new century. This also shifted the focus of the impact factor: For a long time, *Spine* was the highest rated journal in musculoskeletal surgery and medicine worldwide and the “*European Spine Journal*” in Europe. In the last 5–10 years this changed again, as the concept of the impact factor has been increasingly understood by editors of journals and authors.

The impact factor is a widely accepted calculation used to measure the relative importance or impact of science journals. To get an impact factor, a journal has to be indexed in order for the impact factor to be calculated. It seems that journals with larger readership exposure do better and the narrower the subject field of the journal, the better for it. Furthermore, journals with more formal reviews or reference articles do better, and there is a way to increase the impact factor by self-referencing options. Moreover, with time use of impact factors was extended from evaluation of journals, to the evaluation of papers published in those journals, which is not at all the original goal of the metric. This metric can also be influenced by outliers, such as a few articles (or even a single one) cited a huge number of times and boosting the journal’s impact factor way over its “reasonable” value. Therefore, the impact factor calculation does not necessarily have anything to do with the direct reflection of the quality of the journal, but rather expresses a scientific marketing concept.

Finally, the digital age has brought a change in the relation between journal’s impact factor’s and the citation rates of the papers published in those journals, as it appears that digital availability has weakened that relation [1]. Indeed “digital” factors may influence that relationship, for example BMJ has determined that the number of online views for papers on its website was associated with the number of citations [2]. It is therefore highly misleading to judge the quality of a paper (or that of a scientist) based on the impact factor of the journal(s) where article(s) are published [3]. For those reasons new instruments have been developed to alleviate the shortcomings of the impact factor system, which is almost 40 years old. They range from simple extrapolations of the impact factors principles to metrics based on the social media presence of a paper, like the number of Tweets the paper generates in a given period [4].

Some metrics tried to differentiate citations according to the reputations of the citing journal. One of those is the Eigenfactor. Citations in highly ranked journals are weighted and will participate in a greater proportion to the Eigenfactor of a journal than those cited in lower ranking publications [5]. While this metric also has shortcomings it may be considered as more meaningful than the impact factor which is a simple arithmetic measure and which does not take in account the “quality” of citations.

As the pressure for young professionals for their career development got more and more tight, the desire of this younger generation got greater to influence the distribution, the increase and the control of knowledge on one side and to create “à tout prix” new knowledge to advance their careers (“publish or perish”).

The IT revolution, which started to determine more and more facets of our lives, prepared the ground for a massive democratization of knowledge and new possibilities to distribute and communicate knowledge independently of the control by powerful societies, professors and academic institutions—again leading to much more publications.

Certainly the question arises, whether a real increase of additional new knowledge of sufficient quality is happening or whether this is always the same knowledge prepared in many different vessels and forms of communication.

Although the number of papers and articles has massively increased, we cannot ignore the fact that in many of these articles and different journals the same information is available. The creation of substantial new knowledge is obviously slower and less extensive than the possibility to distribute knowledge. This leads today to a burden for the average physician and surgeon to select the new knowledge that really has an impact on patient care and improvement of outcome.

How does a clinician separate the important from the not so important or even useless content? How does a young colleague find his/her orientation and access in research and clinical science? It is impossible for us to read all the information, which is available today in all the different media. The consequences will undoubtedly be that there will be a new function to be covered in the whole medical scientific market: professionals who do nothing else than read and evaluate the widely distributed knowledge and present a summary and appreciation of their reading—a new form of “emminence based medicine” in a world, where we are focusing so much on “evidence based medicine”.

Journals need to understand these changes and this accelerating process to adapt and put their focus of publications to other forms and content of scientific communications. We all may stay attentive to see what is coming and to react appropriately to the changes. The *European Spine Journal* will certainly accept this challenge and will take its place in the driver’s seat.

The so-called “Master Lecture” program which is available on the ESJ-OOT web platform is a further step of the Journal to adapt the communication of established and new knowledge to the needs of our readership.
